# A Student’s Guide to Giant Viruses Infecting Small Eukaryotes: From *Acanthamoeba* to *Zooxanthellae*

**DOI:** 10.3390/v9030046

**Published:** 2017-03-17

**Authors:** Steven W. Wilhelm, Jordan T. Bird, Kyle S. Bonifer, Benjamin C. Calfee, Tian Chen, Samantha R. Coy, P. Jackson Gainer, Eric R. Gann, Huston T. Heatherly, Jasper Lee, Xiaolong Liang, Jiang Liu, April C. Armes, Mohammad Moniruzzaman, J. Hunter Rice, Joshua M. A. Stough, Robert N. Tams, Evan P. Williams, Gary R. LeCleir

**Affiliations:** The Department of Microbiology, The University of Tennessee, Knoxville, TN 37996, USA; jbird9@tennessee.edu (J.T.B.); kbonifer@tennessee.edu (K.S.B.); bcalfee@tennessee.edu (B.C.C.); Tchen18@tennessee.edu (T.C.); srose16@utk.edu (S.R.C.); pgainer@utk.edu (P.J.G.); egann@tennessee.edu (E.R.G.); hheather@tennessee.edu (H.T.H.); jlee175@tennessee.edu (J.L.); xliang5@tennessee.edu (X.L.); jliu36@tennessee.edu (J.L.); amitch51@tennessee.edu (A.C.A.); mmoniruz@tennessee.edu (M.M.); jrice18@utk.edu (J.H.R.); jstough@tennessee.edu (J.M.A.S.); rtams@tennessee.edu (R.N.T.); ewilli99@tennessee.edu (E.P.W.); glecleir@tennessee.edu (G.R.L.)

**Keywords:** giant viruses, nucleocytoplasmic large DNA viruses (NCLDVs), *Mimiviridae*

## Abstract

The discovery of infectious particles that challenge conventional thoughts concerning “what is a virus” has led to the evolution a new field of study in the past decade. Here, we review knowledge and information concerning “giant viruses”, with a focus not only on some of the best studied systems, but also provide an effort to illuminate systems yet to be better resolved. We conclude by demonstrating that there is an abundance of new host–virus systems that fall into this “giant” category, demonstrating that this field of inquiry presents great opportunities for future research.

## 1. Introduction: Defining Giant Viruses

In their editorial introduction to the “Giant Viruses” special issue of Virology, Fischer and Condit [1] stated “It is commonly agreed upon that these are double-stranded DNA (dsDNA) viruses with genome sizes beyond 200 kb pairs, and particles that do not pass through a 0.2-µm pore-size filter”. This definition illustrates the two striking features of giant viruses: their genome and particle size are both larger than has been historically considered for viruses. Beyond their breaking of previous paradigms, how giant viruses are defined remains contentious. Our goal in assembling this synthesis is to provide a “primer” for students of microbiology whom are interested in knowing more about these atypical viruses, and to establish a set of boundaries for their discussion. While not exhaustive, this overview addresses many of the main ideas that, for now, are current within a rapidly expanding field.

**Figure 1:**
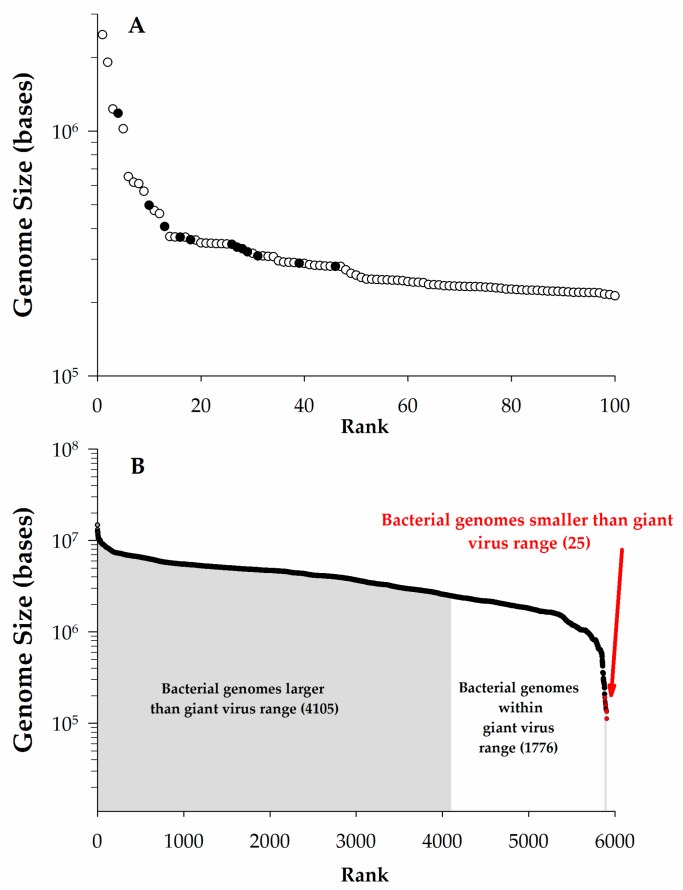
The scale of giant virus genomes. (**A**). Genome size vs. rank plot for the largest 100 complete viral genomes as of January 2016 from National Center for Biotechnology Information (NCBI). Data points noted (●) were previously used in discussion by Claverie et al. [3] to define giants viruses as having genomes > 280 kb, open circles (○) represent additional data; (**B**). Genome size vs. rank order of completed bacterial genomes in NCBI as of January 2016. Sizes are color-coded to match the ranges of giant virus genomes.

Some definitions of giant viruses focus only on genome size with lower limits ranging from undefined [2] to stringent (280 kb or 300 kb) cutoffs [3,4]. Other efforts have focused on the virus particle, suggesting they should be larger than 100 nm [2] or need be easily visible by light microscopy (>300 nm) [5]. One problem with establishing a particular definition for either genome or particle size is that, as additional large viruses are isolated, the rationale may no longer be justified (e.g., Aureococcus anophagefferens virus (AaV), a close phylogenetic relative of *Mimivirus*, is only ~140 nm in diameter) [6]. Indeed, a previous definition proposed a genome minimum of 280 kb due to a notable inflection point in a rank order plot of virus genome size [3]. However, in re-examining the largest 100 complete virus genomes in the National Center for Biotechnology Information’s (NCBI) genome database, this gap is no longer present and a change in slope now occurs at ~400 kb (Figure 1A). This undersampling of giant viruses has resulted in a lack of sufficient information to describe their general characteristics [7,8]. While the vagaries of this definition will fade over time, herein we consider viruses ‘giant’ if their genome is larger than 200 kb. Moreover, this review will focus primarily on giants that infect single-celled eukaryotes.

Using a cutoff of genomic content >200 kb pairs (kbp), ~2.2% (115/5356) of all of the completed virus genomes in NCBI fall within the realm of giants (Figure 1A). To date, all of these giants have genomes consisting of double-stranded DNA: the largest complete genome for other nucleic acid-type viruses is that of the double-stranded RNA (dsRNA) *Dendrolimus punctatus* cypovirus 22 (32.75 kbp) [9]. Perhaps more surprising is that this genome size range for giant viruses overlaps with more than ~one third of the complete prokaryotic genomes in NCBI (Figure 1B), as well as the genome sizes of several small eukaryotes [10]. This includes the smallest free-living archaeon (*Methanothermus fervidus,* 1.2 Mb) and the smallest free-living bacterium (*Candidatus* Actinomarina minuta, estimated ~700 kbp) [11]. While we will not consider them beyond the occasional passing mention in this article, it should be noted that several bacteriophages have genomes exceeding the 200 kbp genome size (see Table 1), and therefore qualify as giants. These phages infect both Gram-positive and -negative bacteria, including cyanobacteria [12,13].

As with observed ranges in genomic size, there is also a wide range of GC content of these viruses relative to the small eukaryotes they infect (Table 1). On average, mobile elements such as phage and plasmids are more AT-rich than their host, but usually by only ~5% [14]. In contrast, Emiliania huxleyi virus (EhV) and AaV, which infect eukaryotic algae, have GC contents that are 24.3% and 38.7% lower than their hosts nuclear genomes, respectively [15,16], while the chloroviruses (freshwater viruses infecting *Chlorella*) have GC contents that are ~21% lower than their host’s nuclear genome. While not a defining feature of all large viruses, this GC difference raises interesting questions concerning the scavenging of nucleotides during the infection cycle. Construction of new viruses is in some cases thought to depend on materials “scavenged” from the host cell, yet in the case of these viruses there would seem to be a discrepancy in terms of what would be available for scavenging. An interest side note to this is that mitochondrial and chloroplast genomes are often observed to have such relative low GC content genomes, similar to these viruses [14,15], implying a potential for scavenged materials from organelles to be important in the construction of new virus particles.

The current size range for giant virus particles varies from our operationally defined ~200 nm to >1500 nm in diameter [5], although as noted, phylogenetic relatives to these giants exist that are only ~140 nm. Indeed, the upper limit of this range is larger than for several bacteria and archaea (Figure 1B), redefining how we think about the relative size of prokaryotes and viruses. These large particle diameters may be needed to house their large genomes (see below), but it has been argued that there are other evolutionary pressures for these virus particles to retain large physical sizes [5]. For example, viruses infecting *Acanthamoeba* are internalized via phagocytosis, and it has been shown that this process works less efficiently on smaller (<600 nm) particles [16]. Additionally, based on standard contact kinetics, a larger particle size may increase the probability of contact between the virus and its host in the environment [17].

**Figure 2:**
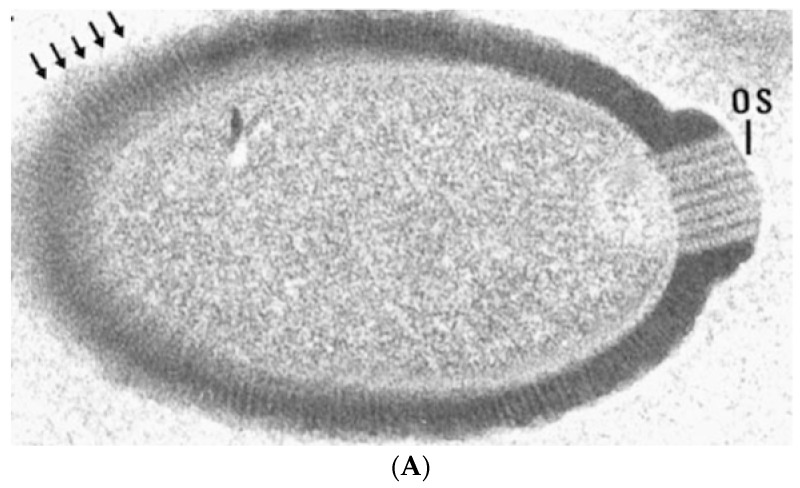

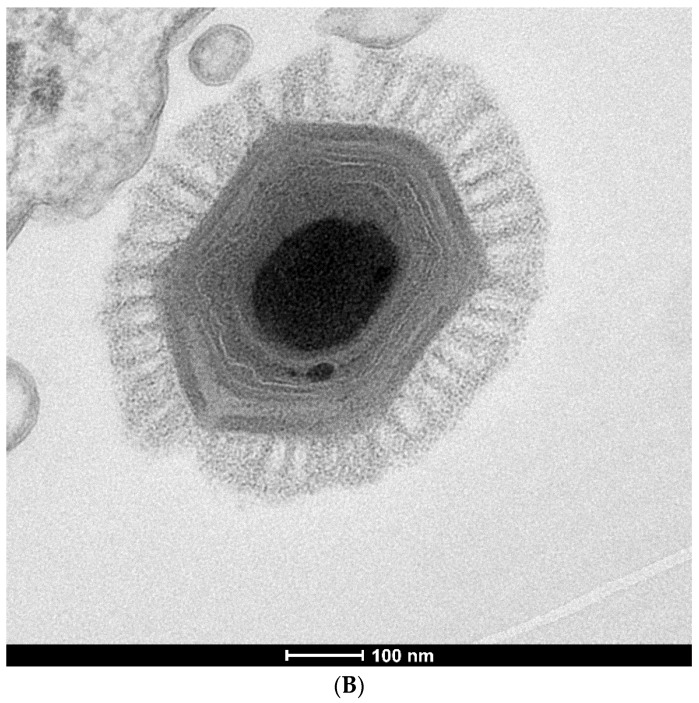
Transmission electron micrographs of giant virus particles. (**A**) *Pithovirus*, as seen in Michel et al. [22]. Originally identified as a KC5/2 parasite, the image shows the electron dense viral wall consisting of perpendicularly oriented fibers or microtubules (arrows), and a marked ostiole (os) located at the apical end of the cell. Reprinted with permission—original magnification at 85,000×; (**B**) *Megavirus chilensis*. Image courtesy of Professors Chantal Abergel and Jean-Michel Claverie.

In addition to a tremendous variation in genome and particle size, giant viruses also have highly diverse morphologies that can be broadly categorized into two groups: ovoid and icosahedral (Figure 2). These morphological differences correspond to the structural proteins that make the virion capsids; icosahedrons are built by homologous β-barrel jelly-roll Major Capsid Proteins (MCPs) with minor capsid proteins acting as scaffolds connecting trisymmetrons and the outer capsid to the inner membrane surrounding the viral genome [18]. In contrast, ovoid viruses encode phylogenetically distant (*Mollivirus)* to unconvincing (*Pandoravirus* and *Pithovirus)* homologs to MCP [19,20,21]. It is unclear how the virion shape provides a selective advantage, since both types have been isolated in similar habitats.

Another mysterious aspect of these giant virus particles are the unique biochemical and morphological features. Virus–host interactions are thought to be facilitated in one of two ways: adsorption to the host cell wall, as is typical of algal host–virus systems [23], or phagocytosis by a protist host. These interactions often involve unique structures. For example, *Mimivirus* and its close relatives (*Megavirus*, *Marseillevirus*, *Lausannevirus*, and *Moumouviru*s) are characterized by proteinaceous fibers anchored to the icosahedron capsid [24,25] that are covered in glycolinkages [26,27,28]. It has been hypothesized that these fibers work in tandem with the large size of the viruses to facilitate phagocytosis, as they appear to have a similar composition to peptidoglycan and thus help mimic a bacterium (indeed, the name *Mimivirus* comes from *“*Mimicking Microbe*”* [29]). Additionally, the fibrous glycoproteins enable viral adsorption to diverse organisms ranging from bacteria and fungi to arthropods [30], implying a potential for both environmental dispersion and an incidental infection strategy in amoeba. *Phycodnaviridae* may also use unique structures to gain access to their host, though their mode of entry is typically by adsorption/injection, as opposed to phagocytosis. For example, the Chlorovirus capsid contains one spike located at a unique vertex of the icosahedral capsid that must be oriented towards the host cell surface to initiate infection [31]. Similarly, *Mimivirus* and its relatives utilize a five-pointed vertex called the ‘stargate’ structure that permits the first step in activating infection. Infection is initiated by fusion of the internal viral lipid membrane to the phagosomal membrane [24,32], which differs from algal viruses that fuse with the host cell membrane. This fusion event is observed in all giant viruses despite differences in structural features or infection strategies [20]. Whether these features are the result of homologous or convergent evolution remains to be determined, though given the breadth of physiological variation in the taxa, conservation of this mechanism is a compelling argument for monophyly.

## 2. Non-Structural Components of the Virion

An anomaly among the giant viruses are several viruses that include EhV*,* which have a lipid envelope outside of the capsid. These viruses include a *Phaeocystis globosa* virus (PgV-07T) [33] and several viruses infecting *Micromonas pussila* [34] which allows for a unique mode of infection and provides protection from environmental stressors [35]. This may be vital to the survival and transmission of these viruses, as they are ingested and transported across blooms by copepods [36]. An additional role of this lipid envelope and its associated proteins is an assumed association with recognition of the host and initiation of infection.

The nucleocytoplasmic large DNA viruses **(**NCLDVs) (described below) package a variety of proteins inside their capsids encoded by either viral or host genomes that are deployed immediately upon infection. For example, the seven proteomes of giant virus particles currently available (below) contain proteins predicted to combat oxidative stress, presumably because viral infections have been shown to generate Reactive Oxygen Species (ROS) that can inhibit viral replication [37]. Interestingly, *Pandoravirus salinus* carries one viral-encoded oxidoreductase, as well as three host-derived proteins predicted to combat ROS [19]. *P. bursaria chlorella* virus-1 (PBCV-1) and the more recently described *Megavirus chilensis* package homologous Cu-Zn superoxide dismutases [38,39]. In *M. chilensis*, this protein is remarkable for having the unique ability to fold and incorporate key metallic cofactors without the aid of chaperone proteins [39]. Additionally, *C. roenbergensis* virus (CroV) and *Mimivirus* both package novel sulfhydryl oxidases that may function in the formation of disulfide bonds [40,41]. These sulfhydryl oxidases as well as other protein disulfide isomerases present in CroV could aid in protein folding or viral entry similar to those found in retroviruses [42,43,44].

## 3. Gauging the Host Range of Giant Viruses in Nature

One concern regarding giant virus isolation using *Acanthamoeba* spp. is that while these are permissive, they may not be the natural hosts. Genomic analyses have been used in an attempt to determine natural hosts. In *Mimivirus*, most of the genes horizontally transferred from eukaryotes originated from amoeba, indicating amoebae are most likely the natural host of *Mimivirus*, but alternative hosts are still possible [45]. Indeed, their unique size and independence from host machinery may allow giant viruses to infect a wide range of hosts, which makes the search for the natural host more challenging. In addition to amoeba, NCLDVs have been reported to infect mice [46] and the symbiotic zooxanthelle of corals [47]. Giant viruses have also been isolated from human blood [48] and have been found in the human virome [49], indicating a potential role in human health (or at least a route of exposure). Indeed, the recent finding of *Acanthocystis turfacea* chlorella virus 1 (ATCV-1) from human oropharyngeal samples is intriguing: subsequent analyses have shown consistency between the presence of these viruses and reduced cognitive function in humans and mice [49].

## 4. Creating (an) Order from the Chaos: The Nucleocytoplasmic Large DNA Viruses

The NCLDV classification was created to define a monophyletic group of families that, when initially conceived, included *Asfarviridae*, *Phycodnaviridae*, *Poxviridae*, and *Iridoviridae* [50]. The rationale for this grouping was based on a conserved core of (1) nine genes hypothesized as representative of a common NCLDV ancestor and (2) a total of 22 more genes found in at least three of the four constituent viral families. The name is a reference to the replicative strategies of the included families as they replicate in both the nucleus and cytoplasm (phycodnaviruses, asfarvavirus and iridovirus) [51,52,53], or totally within the cytoplasm (poxviruses) [54]. A NCLDV ancestor has been hypothesized to have originated early in evolutionary history, possibly contemporaneously with early eukaryotic evolution, as suggested by the broad host range of NCLDV members [55]. However, the nature of this ancestral NCLDV remains unclear. Due to non-orthologous displacement of core genes [55] and potential reductive evolution [5] it is especially difficult to estimate the approximate genome size of any common ancestor and whether it would qualify as a giant virus when compared with modern giants. Indeed given theories on genome size variability, such as the genomic accordion [56,57], it is likely that predecessors of a variety of genome sizes existed. Moreover, it has been argued that mobile genetic elements encoded by virophages and transpovirions may have contributed significantly to the size of the NCLDV genome [58,59]. Therefore, the ancestral NCLDV may have been much smaller in genome size than modern representatives, and the mechanism by which it expanded its genome may have resulted in the wide range of genome sizes seen in current NCLDV members [60].

The NCLDV classification is not without its shortcomings. As new members are added to the group, the “nucleocytoplasmic” distinction of replicative strategies becomes less useful due to the increasing diversity of virion production. Many NCLDV families utilize a nucleocytoplasmic route for replication, including *Asfarviridae* [52], *Iridoviridae* and *Ascoviridae* [53], *Phycodnaviridae* [51], and *Pandoraviridae*. Other families, like *Poxviridae* [54], *Mimiviridae* [61], *Marseilleviridae* [62] and *Pithoviridae* [5], begin and complete their replication cycles exclusively in the cytoplasm, encoding the replication and transcription machinery necessary to produce virions without nuclear involvement. From a taxonomic perspective, the NCLDV group does not follow the naming conventions of (and is not recognized by) the International Committee on Taxonomy of Viruses (ICTV), as the classification lacks context within a larger hierarchy. To rectify this, Colson et al. proposed to reclassify NCLDVs within the new viral order *Megavirales* [63,64] based on the presence of conserved ancestral genes and a large icosahedral capsid composed of a homologous β-barrel jelly roll protein. This classification scheme, however, excludes the *Poxviridae* and *Ascoviridae*, [65] as well as *Pandoravirus*, *Pithovirus* and *Mollivirus*. In addition, the *Megavirales* classification required the capacity to assemble viral factories within the cytoplasm of host cells [62,66,67,68,69], a feature found in RNA viruses [70] but not seen in DNA viruses outside of the NCLDV group [64]. Currently (as of December 2016), the *Megavirales* is not considered a classification by the ICTV.

Most recently, the NCLDV genome size range has expanded to include genomes from 100 kb to 2.77 Mb encoding from 110 to 2556 genes [19,60]. The ten groups of NCLDV (*Phycodnaviridae*, *Poxviridae*, *Asfarviridae*, *Ascoviridae*, *Iridoviridae*, *Mimiviridae*, *Marseilleviridae*, *Pandoraviridae*, *Pithovirus*, and *Mollivirus*) infect a broad spectrum of hosts. In keeping with the NCLDV group’s high degree of variability regarding particle size and host range, these viruses also display varying degrees of reliance on host metabolism and machinery, resulting in a limited number of highly conserved or “core” genes (e.g., see [71]). Yet despite these variances in NCLDV traits, common ground does exist. There are genes conserved amongst all available NCLDV genomes that are crucial for viral production or virion structure, such as the D5R packaging ATPase, D13L major capsid protein, and the B family DNA polymerase.

Comparative analyses of the genes conserved amongst different giant virus families has historically supported the monophyletic nature of the NCLDV group, and recent efforts to determine the clusters of orthologous groups (COGs) for giant viruses support their monophyly [72]. The conserved genes further provide potential markers that might be used in the discovery of novel NCLDVs and the determination of phylogenetic relationships between more closely related taxa [60]. For example, Moniruzzaman and colleagues [73] demonstrated an expanded level of diversity of the algal-specific members of the *Mimiviridae* by targeting the conserved MCP gene in this clade. However, this approach has its limits; the three recently discovered representatives of *Pandoravirus* lack the major capsid protein and the D5R helicase, as well as a number of other core NCLDV genes [19]. Indeed only 17 of the 49 inferred ancestral NCLDV genes were found in at least one of the *Pandoravirus* genomes, calling into question their inclusion in the giant virus clade despite their particle and genome size [74].

## 5. Viruses as a Possible Fourth Domain of Life

Initially viruses were defined by their intrinsic filterability away from cellular life forms [75,76,77], a definition subsequently refined to include their lack of ribosomes, a host-dependent metabolic strategy, and replication by means other than binary fission [78]. That the unique capabilities of NCLDVs still fit well within the latter definition, after fifty years of discovery and scientific scrutiny, highlights a fundamental difference between cellular organisms and viral particles. However, giant viruses do challenge these distinctions. Independent of their size, which invalidates the informal 0.2-μm filter cutoff, NCLDVs are remarkably cell-like in virion structure and gene content. In addition to their protein coat, membrane, and genome, *Mimivirus* and *Marseillevirus* particles contain messenger RNA molecules, making them the only viruses, to date, that contain both types of nucleic acid [79]. Moreover, several viruses encode genes involved in translational processes, such as varying numbers of aminoacyl-transfer RNA (tRNA) synthetases [69,80]. Indeed, we hypothesize these proteins may be useful in overcoming differences in GC content seen between some viruses and hosts (Table 1), but this has yet to be empirically demonstrated.

The discovery of translational machinery (including that mentioned above) encoded in select virus genomes allows for comparisons to traditionally “cellular” functions normally associated with the three domains of life. Sequence alignments comparing multiple genes involved in DNA replication and repair, transcription, and translation shared between cellular organisms and NCLDVs appear to show deeply branching relationships as ancient as the domain Eukarya. It was subsequently hypothesized that giant viruses evolved from a cellular common ancestor belonging to a currently extinct fourth domain of life, unique from Bacteria, Archaea, and Eukarya [63,81]. Seemingly in support of this hypothesis is the abundance of coding sequences (ORFans) in giant viral genomes with no known homologues in the other domains.

These ideas have proven somewhat controversial, as direct sequence comparison of genes conserved among cellular organisms with virus-encoded homologs is problematic. As selective pressures on similar genes within viruses and their hosts are likely different, accelerated sequence divergence in viruses may exaggerate their perceived distance from the derived gene [82]. Subsequent alignments accounting for compositional heterogeneity and homoplasy place giant virus genes with eukaryotes [83]. While it has been countered that giant virus genes do not evolve more quickly than their cellular counterparts, this has yet to be demonstrated outside of a single example within *Marseilleviridae* [5,84]. Indeed, an overabundance of viral open reading frames (ORFs) without known homologues is not a problem unique to giant viruses [85]. These observations and others have led to the alternative hypothesis: gene content within the different NCLDV families suggests that their genomes have been built up from smaller viruses over time, rather than by loss of unnecessary genes by an ancient cellular ancestor [72]. As some of the current NCLDVs replicate in phagotrophs like *Acanthamoeba* and *Cafeteria roenbergensis*, it was hypothesized that smaller viruses may incorporate genetic material from other organisms phagocytosed by the host.

## 6. Giant Viruses in the Environment

While surveys are not yet exhaustive, giant viruses appear to be found in all environments. Since the discovery of *Mimivirus* from a water cooling tower [86], giant viruses have been found in locations where amoebae normally thrive, including seawater, soil, aerosols, and man-made aquatic environments such as sewage, fountains and air conditioners [87], in addition to harsh, unexpected ecosystems such as permafrost [20]. Lastly, giant viruses or their DNA sequences have been observed in animals such as dinoflagellate-associated coral [47], arthropods, and humans [49,88].

A powerful tool in the identification of putative new viruses are environmental metagenomic studies (Table 2), though most have not focused specifically on giant viruses until recently [21]. Current research suggests giant viruses only comprise a small percentage of viruses (<1%) in most samples. However, virus densities can fluctuate based on contact with their host: for example, *Chlorella* viruses are much more abundant when their hosts, normally sequestered as endosymbionts of *Paramecium bursaria*, are made available as a consequence of predatory activity on the *Paramecium* [89]. Regardless, it is clear some families tend to be more common than others: in marine metagenomics samples *Phycodnaviridae*-related sequences were found to be highest in abundance, followed by *Mimiviridae* [90,91]. It is also clear that these viruses are persistent: the discovery of 30,000-year-old *Mollivirus* particles in permafrost suggests that giant viruses can survive, under the correct conditions, for long periods of time [21]. When combined with other tools such as flow cytometry sorting of either individual particles [92] or infected hosts [93], these new approaches will begin to shed significant light on the natural diversity of these populations.

To date, much of the focus on giant viruses has been on their genomics rather than their influence on the environments in which they persist. Several large, dsDNA viruses including EhV [97], PgV [98], AaV [6,97] and *Heterosigma akashiwo* virus (HaV) [99] are associated with algal blooms, although only a few have been directly shown to infect and lyse the phytoplankton involved with the bloom in situ [97,100]. Algal blooms occur on large geographical scales and result in significant influxes of atmospheric carbon into the world’s oceans. Viruses, particularly bacteriophages, are known drivers of dissolved organic matter (DOM) release back into the environment via a process known as the “viral shunt” [101,102]. With the large biomass of algae associated with these blooms, virus-mediated collapse by giant viruses may also be an important driver of dissolved and particulate organic matter release. Giant viruses that infect algae may be likened to bacteriophages in terms of participating in the viral shunt, and the release of nutrients back into the environment may be an important part of the ecological cycle in aquatic systems [102].

A recent estimate suggested that giant viruses available in culture were infectious to at least 22 different algal species [103]. Globally, it has been proposed that there are more than 350,000 algal species [104]. Given the possibility that all algae may be infected with one or more viruses [105,106], the possibility of a collection of unknown giants remains very real, and indeed molecular data point to at least a broad diversity within the known groups [107,108]. Building on the above, it is clear from a survey of the literature that researchers identified candidate protist-giant virus systems well before *Mimivirus* was documented (Table 3). In the late 1960s and early 1970s, the expanded availability of transmission electron microscopes to researchers resulted in a series of observations concerning the presence of large virus-like particles inside algal cells [107,109]. In many cases, these virus–host systems have been largely ignored by the scientific community, creating a broad spectrum of opportunities for researchers to begin to cultivate these plankton in an effort to isolate and characterize new giant viruses. Given the expansive putative host-range that has been observed, it is likely that many of these viruses could fill in knowledge gaps concerning the diversity and potential function of these particles. Indeed, one example of how new hosts can be used to discover new viruses are the *Faustovirus*, recently discovered using *Vermamoeba* (a protist found in both humans and natural systems) as a screen [110]: unique to these viruses is a collection of genes three times larger than the other members of the *Asfarviridae* family.

And while it is obvious that there is a dearth of knowledge concerning giant viruses that infect algae in the environment, there is an even larger knowledge gap regarding giant viruses infecting heterotrophic eukaryotes. The most studied of these viruses is CroV, which infects the heterotrophic grazer *Cafeteria roenbergensis* [111]*.* Given this organism is a grazer of primary producers it is possible that infection of this organism by CroV could have effects on lower trophic level organisms. It has been shown that grazing can be an important driver of algal bloom decline [112], so it stands to reason that the effects giant viruses have on mixo/heterotrophic-plankton are critical to understanding bloom dynamics. Almost no information, at this time, is available to discuss the impacts of these infections, but they will most likely result in interesting discoveries and further our understanding of how giant viruses alter the microbial food web.

## 7. Intimate Interactions with the Host: Eco-Evolutionary Consequences

Only recently have we come to appreciate the possibility of gene transfer between giant viruses and their hosts. A large proportion of giant virus genes comes from diverse sources, including from their eukaryotic hosts [127]. In EhV, seven genes involved in sphingolipid biosynthesis pathway were putatively transferred from the host algae [128]. Upon infection, the host sphingolipid biosynthesis pathway is downregulated concomitant with the upregulation of the corresponding viral genes, leading to increased production of viral glycosphingolipids (vGSLs) [129]. EhV particles are covered by vGSLs, and this unique lipid molecule ultimately induces programmed cell death (PCD) in infected hosts [130].

A genome wide phylogenetic study of AaV identified a number of genes having their highest phylogenetic affinity to host (*Aureococcus*) homologs, [71]. This agrees with observations made by earlier studies on several other giant viruses [127,131]. While gene acquisition may be one of the evolutionary strategies of giant viruses, how these genes confer ecological advantages remains largely unknown. As the vast majority of viruses harbor streamlined genomes with few genes, the enormous genetic resource of giant viruses poses a paradox in terms of energetic cost of replication. Closer inspection of a number of sequenced eukaryotic genomes revealed a large number of genes originated from giant viruses [132,133]. In a recent study, large genomic islands, putatively derived from both giant viruses and a virophage, were found in *Bigelowiella natans*, a Cryptomonad algae [134]. In another study, “core” genes from giant viruses were detected in eight protists and a metazoan (*Hydra magnipapillata*) genome [132]. Remarkably, a 400-kb region in the *H. magnipapillata* was putatively identified to be of viral origin [132]. Major capsid gene phylogeny indicated the genes were likely from a *Mimiviridae* family member. Giant virus particles and marker genes have also recently also been observed associated with zooxanthellae from the genus *Symbiodinium*, a dinoflagellate typically found closely associated with corals [47]. Giant virus-like genes were also found in several other protists [133,135] and some plant genomes, namely *Physcomitrella patens* and *Selaginella moellendorffii* [136]. The role of giant virus-derived genes in host remain an open question.

Host–virus interactions result in an evolutionary arms race—leading to the emergence of new diversity in the host and virus population [137]. Hosts of giant viruses have evolved a variety of defense mechanisms against giant viruses. An elegant example is the ‘Cheshire cat’ strategy adopted by *Emiliania huxleyi* [138]. The diploid calcified cells of *E. huxleyi* are susceptible to EhV infection, while the haploid stage is ‘invisible’ to infection. It has been suggested that during the decline of the Brown tide blooms, a virus-resistant population of the *Aureococcus* persists, maintaining a relatively high abundance of *Aureococcus* even after the demise of the bloom [73,97].

## 8. Virophage

Another interesting characteristic of some giant viruses is their susceptibility to infection by other bioactive particles, termed “virophage”. The first virophage to be isolated was named Sputnik [59], which replicates within the viral factory used by *Mamavirus* within *Acanthamoeba castellanii*. Because of this, Sputnik only replicates within *A. castellanii* co-infected with Mamavirus. Infection by the virophage causes abnormal capsid structure of *Mamavirus*, increasing capsid size and causing abnormal fiber localization on its surface, suggesting a parasitic relationship between the two [59]. Co-incubation of Sputnik and *Mamavirus* decreased infective *Mamavirus* particle titers by approximately 70% and increased the survival rate of the *A. castellanii* [59]. Similar virophages have been found infecting other giant viruses as well [139,140,141,142]. The discovery of “viruses that infect viruses” has strengthened the argument that viruses are living entities [143]. Some classes of *Mimivirus* appear to have developed a CRISPR-CAS-like system suggested to combat these virophages, called the *Mimivirus* virophage resistant element (MIMIVIRE) [144]. Interestingly, a number of genes homologous to those in the MIMIVIRE system are present in other giant viruses, suggesting that the MIMIVIRE-like defense systems might not be exclusive to *Mimivirus* [134,145]*.* Other interpretations, however, are questioning these conclusions [146]*.* Much has yet to be learned in these systems, but virophages may act like both ‘provirophage’ and ‘provirus’, depending on the genomic context at multiple levels.

## 9. Conclusions

The discovery of *Mimivirus* has driven both the nascence and evolution of a new area of scientific inquiry. Giant viruses are now the topics of evolutionary, ecological and biotechnological inquiries. Moreover, broad-scale efforts to identify new virus–host systems, ranging from classic culture-based approaches to newer bioinformatics efforts to link viruses and their hosts [147] will soon provide a larger data base of information concerning the key features of these novel virus particles. Indeed, a survey of older literature (Table 3) clearly demonstrates that there are many virus–host systems that have been observed but are yet to be isolated and characterized. Moving forward, there is little doubt that the study of giant viruses will shed new light not only on virus–host relationships, but also on key evolutionary processes including the natural occurrence rates of transduction and horizontal gene transfer.

## Figures and Tables

**Table 1:** Comparison of host and viral genome size and GC content. All data was collected from the NCBI repository.

Giant Virus	Size Virus (Mb)	Virus GC (%)	ORFs *	Accession	Host	Size Host (Mb)	Host GC (%)	Host-Virus Genome Size	Host-Virus GC	Accession
*Pandoravirus salinus*	2.5	61.7	2541	NC_022098.1	*Acanthamoeba castellanii*	46.7	58.3	18.9	−3.4	AHJI00000000.1
*Pandoravirus dulcis*	1.9	63.7	1487	NC_021858.1	*A. castellanii*	46.7	58.3	24.5	−5.4	AHJI00000000.1
*Acanthamoeba polyphaga mimivirus*	1.2	28.0	1018	NC_014649.1	*A. polyphaga*	120.4	59.3	102.0	31.3	CDFK00000000.1
*Acanthamoeba polyphaga moumouvirus*	1.0	24.6	915	NC_020104.1	*A. polyphaga*	120.4	59.3	118.1	34.7	CDFK00000000.1
*Mollivirus sibericum*	0.7	60.1	523	NC_027867.1	*A. castellanii*	42.0	58.4	64.6	−1.7	AHJI00000000.1
*Pithovirus* *sibericum*	0.6	35.8	467	NC_023423.1	*A. castellanii*	42.0	58.4	68.9	22.6	AHJI00000000.1
Emiliania huxleyi virus 86	0.4	40.2	478	NC_007346.1	*Emiliania huxleyi*	167.7	65.7	409.0	25.5	AHAL00000000.1
*Marseillevirus marseillevirus*	0.4	44.7	457	NC_013756.1	*A. polyphaga*	120.4	59.3	325.5	14.6	CDFK00000000.1
Aureococcus anophagefferens virus	0.4	28.7	384	NC_024697.1	*A. anophagefferens*	56.7	69.5	153.1	40.8	NZ_ACJI00000000.1
*Melbournevirus*	0.4	44.7	403	NC_025412.1	*A. castellanii*	42.0	58.4	113.6	13.7	AHJI00000000.1
*Paramecium bursaria* Chlorella virus NY2A	0.4	40.7	411	NC_009898.1	*Chlorella variabilis* NC64A	46.2	67.1	124.8	26.4	ADIC00000000.1
Brazilian marseillevirus	0.4	43.3	491	NC_029692.1	*A. castellanii*	42.0	58.4	116.7	15.1	AHJI00000000.1
*Lausannevirus*	0.4	42.9	444	NC_015326.1	*A. castellanii*	42.0	58.4	120.1	15.5	AHJI00000000.1
*Ectocarpus siliculosus* virus 1	0.3	51.7	240	NC_002687.1	*Ectocarpus siliculosus*	195.8	53.5	575.9	1.8	CABU00000000.1
*Paramecium bursaria* Chlorella virus AR158	0.3	40.8	366	NC_009899.1	*C. variabilis* NC64A	46.2	67.1	135.8	26.3	ADIC00000000.1
*Paramecium bursaria* Chlorella virus 1	0.3	40.0	376	NC_000852.5	*C. variabilis* NC64A	46.2	67.1	139.9	27.1	ADIC00000000.1
Micromonas pusilla virus 12T	0.2	39.8	265	NC_020864.1	*Micromonas pusilla*	22.0	65.9	104.6	26.1	NZ_ACCP00000000.1
Sample Bacteriophage										
*Bacillus* phage G	0.5	29.9	694	NC_023719.1	*Bacillus megaterium*	5.3	38.1	10.7	8.2	NZ_CP009920.1
*Prochlorococcus* phage P-SSM2	0.3	35.5	335	NC_006883.2	*Prochlorococcus marinus*	1.8	36.4	7.0	0.9	NC_005042.1
*Ralstonia* phage RSL1	0.2	58.0	345	NC_010811.2	*Ralstonia solanacearum*	5.6	66.5	24.3	8.5	NC_003295.1
*Sinorhizobium* phage phiN3	0.2	49.1	408	NC_028945.1	*Sinorhizobium meliloti*	3.7	62.7	17.4	13.6	NC_003047.1
*Pseudomonas* phage EL	0.2	49.3	201	NC_007623.1	*Pseudomonas aeruginosa*	6.3	66.6	29.8	17.3	NC_002516.2

* ORF = Open reading frame

**Table 2:** Comparison of giant virus reads to total viral reads in shotgun metagenomic studies from different environments.

Environment	Location	Abundance	Total Reads	Most Common Virus Families Present	Source
Marine	Indian Ocean	0.3%–1.4%	N/A	*Mimiviridae*, *Phycodnaviridae*	[91]
Antarctic soil	Antarctica	2.82%–7.71%	123/1595-177/6264	*Mimiviridae*, *Phycodnaviridae*	[94]
Coral	USA	1.2%	744/60485	*Mimiviridae*, *Phycodnaviridae*	[95]
Human (respiratory system)	Sweden	0.00002%	2/111931	*Mimiviridae*	[96]

**Table 3:** A chronological list of organisms shown in the literature to contain viruses consistent with the giant virus size class.

Year	Organism	Particle Size	References
1970	*Aphelidium* sp. (fungal parasite of algae)	190–210 nm	[113]
1972	*Oedogonium* spp. “L” (Chlorophyceae)	240 nm	[114]
	*Chorda tomentosa* (Phaeophyceae)	170 nm	[115]
1973	*Ectocarpus* sp.; *Ectocarpus fasciculatus* (Phaeophyceae)	150 nm, 170 nm	[116,117]
	*Aulacomonas submarina* (Chlorophyceae)	200–230 nm	[118]
1974	*Pylaiella littoralis* (Phaeophyceae)	130–170 nm	[119]
	*Pyramimonas orientalis* (Prasinophyceae)	200 nm	[120]
1975 ^†^	*Chara corallina (*Charophyceae*)*	18 nm × 532 nm	[121]
1978	*Sorocarpus uvaeformis* (Phaeophyceae)	170 nm	[122]
1979	*Gymnodinium uberrimum* (Dinophyceae)	385 nm	[123]
	*Mallomonas* sp. (Synurophyceae)	175 nm	[123]
1980	*Uronema gigas* (Chlorophyceae)	390 nm	[124]
1984	*Paraphysomonas* *corynephora* (Chrysophyceae)	150–180 nm, 270–300 nm	[125]
1993	Various Phaeodarian food vacuoles	300–750 nm	[126]

^†^ Although in length this virus qualifies as a giant, its rod shaped morphology is more consistent with Tobacco mosaic virus than any member of the *Mimiviridae*.
